# Nucleoside Analogs and Nucleoside Precursors as Drugs in the Fight against SARS-CoV-2 and Other Coronaviruses

**DOI:** 10.3390/molecules26040986

**Published:** 2021-02-13

**Authors:** Nicola Borbone, Gennaro Piccialli, Giovanni Nicola Roviello, Giorgia Oliviero

**Affiliations:** 1 Department of Pharmacy, University of Naples Federico II, Via Domenico Montesano 49, 80131 Naples, Italy; nicola.borbone@unina.it (N.B.); picciall@unina.it (G.P.); 2 Institute of Biostructures and Bioimaging-CNR 1, Via Mezzocannone 16, 80134 Naples, Italy; 3 Department of Molecular Medicine and Medical Biotechnologies, University of Napoli Federico II, Via Sergio Pansini 5, 80131 Naples, Italy; golivier@unina.it

**Keywords:** coronavirus, SARS-CoV-1, SARS-CoV-2, MERS-CoV, nucleoside drugs, remdesivir, ribavirin, favipiravir, molnupiravir, sofosbuvir

## Abstract

Coronaviruses (CoVs) are positive-sense RNA enveloped viruses, members of the family Coronaviridae, that cause infections in a broad range of mammals including humans. Several CoV species lead to mild upper respiratory infections typically associated with common colds. However, three human CoV (HCoV) species: Severe Acute Respiratory Syndrome (SARS)-CoV-1, Middle East Respiratory Syndrome (MERS)-CoV, and SARS-CoV-2, are responsible for severe respiratory diseases at the origin of two recent epidemics (SARS and MERS), and of the current COronaVIrus Disease 19 (COVID-19), respectively. The easily transmissible SARS-CoV-2, emerging at the end of 2019 in China, spread rapidly worldwide, leading the World Health Organization (WHO) to declare COVID-19 a pandemic. While the world waits for mass vaccination, there is an urgent need for effective drugs as short-term weapons to combat the SARS-CoV-2 infection. In this context, the drug repurposing approach is a strategy able to guarantee positive results rapidly. In this regard, it is well known that several nucleoside-mimicking analogs and nucleoside precursors may inhibit the growth of viruses providing effective therapies for several viral diseases, including HCoV infections. Therefore, this review will focus on synthetic nucleosides and nucleoside precursors active against different HCoV species, paying great attention to SARS-CoV-2. This work covers progress made in anti-CoV therapy with nucleoside derivatives and provides insight into their main mechanisms of action.

## 1. Introduction

Coronaviruses (CoVs) are enveloped positive-sense single-stranded RNA viruses belonging to the family Coronaviridae, causing infections in avian species, mammals, and, among these, humans [1,2,3,4,5,6]. Human coronaviruses (HCoV) are believed to be of zoonotic origin, and their infections mainly lead to respiratory diseases [7,8,9]. In particular, HCoV-229E, HCoV-OC43, HCoV-NL63, and HCoV-HKU1 cause the mild seasonal symptoms of the common cold [10,11]. However, three HCoV species responsible for the onset of life-threatening respiratory events emerged in the last two decades: Severe Acute Respiratory Syndrome (SARS)-CoV-1, Middle East Respiratory Syndrome (MERS)-CoV, and SARS-CoV-2 [12,13,14,15]. Human infection by SARS-CoV-2 is at the origin of the current COronaVIrus Disease 19 (COVID-19) pandemic. Interestingly, SARS-CoV-1 and MERS-CoV are more lethal but less transmissible than SARS-CoV-2, to which they are closely related [16]. There is clearly an urgent need for mass immunization and specific treatments for these HCoV-associated pathologies. CoV infection starts with the specific molecular recognition between the CoV spike (S) protein and host-specific receptors exposed on the surface of the target cells [17,18,19,20,21]. These have been identified for several CoVs and represent the primary molecular targets for anti-CoV strategies [22]. Human aminopeptidase N (APN) is involved in the infection by HCoV-229E; 9-O-acetylated sialic acid (9-O-Ac-Sia) receptor for HCoV-OC43 and HCoV-HKU1; angiotensin-converting enzyme 2 (ACE2) for HCoV-NL63, SARS-CoV-1, and SARS-CoV-2; dipeptidyl peptidase 4 (DPP4) for MERS-CoV [23,24]. Intracellularly, CoVs replicate their RNA and produce the viral proteins required for the assembly of new viral particles [25]. While five out of the seven HCoVs are usually associated with mild upper respiratory infections, MERS-CoV and SARS-CoV-1 and 2 can lead to lethal events [26]. In particular, the new SARS-CoV-2, first emerging in China at the end of 2019 [26], can provoke severe pneumonia, and being easily transmissible, it rapidly spread worldwide leading the World Health Organization (WHO) to declare COVID-19 a pandemic in March 2020 [27]. Currently, there have been more than two million deaths due to COVID-19 (2,239,418 as found in Worldometers.info [28] accessed on 1 February 2021), with enormous consequences for public health and the global economy [29,30,31]. While the whole world is fighting against COVID-19 and waits for a global and effective vaccination, the scientific community is devoting immense efforts to develop effective drugs for the immediate treatment of SARS-CoV-2 infection. Due to the urgent need for such a pharmacological treatment, drug repurposing [32,33] is one of the most common approaches. In this context, nucleobase-containing synthetic molecules [34,35,36,37,38,39,40] and modified nucleosides [41,42,43,44,45,46] are attracting significant interest for their antiviral activity [47,48,49]. In particular, nucleoside-mimicking analogs [50], as well as nucleoside precursors [51,52], being able to inhibit the growth of viruses, play a pivotal role in the search of effective therapies for HCoV infectious diseases [53,54].

## 2. Human Coronaviruses

Presently, seven HCoVs are known and described in the scientific literature [55]. Besides the well-known potentially lethal SARS-CoV-1, MERS-CoV, and SARS-CoV-2, the common human coronaviruses HCoV-229E, HCoV-NL63, HCoV-OC43, and HCoV-HKU1 identified in the last few decades were classified into two CoV genera: Alphacoronavirus and Betacoronavirus [56]. 

HCoV-229E and HCoV-NL63, belonging to the genus Alphacoronavirus [57], are genetically related to each other and are responsible for about 5% of all respiratory infections in hospitalized children [58]. Both HCoV-OC43 and HCoV-HKU1, of the genus Betacoronavirus [59], are ‘common cold’ viruses widely circulating worldwide, with associated severity of respiratory symptoms being documented only in rare cases [60]. Even though these HCoVs do not cause severe clinical symptoms in most patients, HCoV 229E and OC43 can provoke pneumonia [61,62], while HCoV-NL63 and HCoV-HKU1 infection lead in some cases also to bronchiolitis and croup [63,64].

### Human Coronaviruses Causing Lethal Pneumonia: SARS-CoV-1, MERS-CoV, and SARS-CoV-2

The first HCoV-recognized pandemic, known as ‘Severe Acute Respiratory Syndrome’ (SARS), was caused by SARS-CoV-1 in China in 2002–2003 [65,66]. It spread to 29 countries worldwide, infecting over 8,000 people, with a 10% death rate [67]. Another deadly coronavirus emerged about ten years later, leading to the so-called ‘Middle East Respiratory Syndrome’ (MERS) and was indicated, thus, MERS-CoV [68]. After its outbreak in Jordan among hospital workers, it spread to Saudi Arabia in 2013–2014 [69]. MERS occurs predominantly in male individuals, while the same is not seen for SARS. The main clinical symptoms of both SARS and MERS include fever, cough, shortness of breath, and respiratory illness, with MERS being associated with more severe pneumonia and a higher mortality rate of up to 35% vs. the already-cited 10% of SARS [70]. Both MERS and SARS affect mainly adult individuals with a median age range of 39–50 years [62]. SARS-CoV-2 is closely related to SARS-CoV-1, with which it shares about 79% genome sequence identity, and is responsible for the current COVID-19 [71].

Like SARS-CoV-1 and MERS-CoV, SARS-CoV-2 belongs to the genus Betacoronavirus, but while the two SARS-CoVs are further classified into the Sarbecovirus subgenus, MERS-CoV belongs to the Merbecovirus subgenus [72]. Globally, the WHO estimated a mortality rate due to COVID-19 of 3.4%, though there is no definitive consensus on this estimate [73]. Whatever the mortality rate of SARS-CoV-2, one of the most worrying aspects of this virus is that it spreads easily and rapidly, having reached in a few months hundreds of countries infecting more than 100 million individuals.

## 3. Prophylaxis and Therapy of HCoV Diseases

Before 2002, the only known HCoVs were the common human coronaviruses associated with ‘common cold’ symptoms mentioned above. Since most people with an illness caused by them usually recovered spontaneously, there was typically no need for any drugs other than aspirin to relieve the cold-associated symptoms. Conversely, the critical clinical conditions often observed in patients affected by the highly pathogenic MERS-CoV, SARS-CoV-1, and, especially, SARS-CoV-2 recalled the urgency of developing vaccines and antiviral treatments for HCoV infections. This research theme was previously almost ignored by pharmaceutical companies and, in our opinion, should not be abandoned by the scientific community even when the COVID-19 emergency will be over. In SARS-CoV-1 infection, scientists undertook initial vaccine studies, but the obtained candidates presented severe complications such as immune disease insurgence in treated animals [74]. The research for other SARS vaccines was discontinued not only for the difficulties encountered, but mainly because SARS-CoV-1 vanished [74]. Owing to the pharmacological strategies adopted by physicians for SARS patients, these were essentially empirical and involved repurposed immunomodulatory and antiviral drugs, such as corticosteroids, lopinavir/ritonavir, and ribavirin [32,33]. Concerning MERS, despite several efforts to search for effective vaccines, antibodies, and drugs, no conclusive results were achieved. Repurposed drugs used with some success for MERS include again lopinavir/ritonavir and ribavirin [75,76]. After this premise, it appears clear how the lack of any useful vaccine and drug against SARS-CoV-1 and MERS-CoV was reflected in the current crisis, considering the relatively close relationship between SARS-CoV-1 and SARS-CoV-2 genomes, as well as the conserved nature of MERS and SARS-CoV-2 proteins [71]. Fortunately, academic institutions and pharmaceutical companies have lately developed some promising vaccine candidates against SARS-CoV-2. Among them, the Pfizer-BioNTech (BNT162b2) [77], the Moderna (mRNA-1273) [78], and the Oxford University/AstraZeneca (ChAdOx1-S) [79] vaccines were authorized for prophylaxis of COVID-19, while several others are currently in late-stage clinical testing [80]. Vaccines may represent a medium/long-term solution to the current pandemic, but short-term solutions such as pharmacological treatments against SARS-CoV-2 remain urgently needed. Presently, SARS-CoV-2 infection therapy includes immunomodulatory drugs, plasma from individuals recovered from COVID-19, and several pharmacological treatments [81]. In this regard, despite numerous repurposed drugs being tested, only the nucleoside analog remdesivir has been officially approved by the American Food and Drug Administration (FDA) agency to date [82].

## 4. Protein Targets for Anti-HCoV Pharmaceutical Strategies

Amongst the coronavirus targets that were studied, or are currently being investigated, in the fight against the three most pathogenic HCoVs, particular relevance is given to the spike (S) protein [17,18,19,20,21], RNA-dependent RNA-polymerase (RdRp) [83,84,85,86,87], papain-like protease (PL^pro^) [88,89] and main protease (M^pro^, 3CL^pro^) [90,91]. In particular, this latter, which proteolytically cleaves the polyproteins to functional proteins essential for viral replication, occupies a special place in pharmaceutical research [92]. Hence, the frequently-reported administration of potential M^pro^ inhibitors like lopinavir and ritonavir to SARS, MERS, and COVID patients [76,93,94], even though there is no agreement on the real efficacy of this cocktail therapy, especially in the case of the current pandemic [94]. RdRp is a protein involved in SARS-CoV-2 replication, considered to be conserved within RNA viruses [95]. Targeting the RdRp by antiviral drugs could be a potential therapeutic option to inhibit coronavirus RNA polymerization and, consequently, viral replication. Since remdesivir [82], the only FDA-approved drug for COVID-19 available to date, is believed to inhibit SARS-CoV-2 RNA polymerase competing with natural nucleotide triphosphates for incorporation into growing viral RNA, this aspect attracts interest not only on this drug but also on other analogs of nucleosides and nucleoside precursors with similar RdRp inhibitory activity.

## 5. Synthetic Nucleoside Precursors for HCoV Disease Therapy

**Figure 1:**
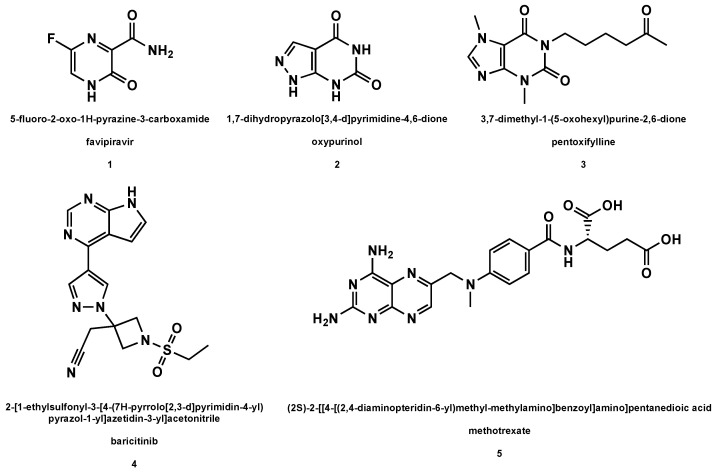
Structures of the nucleoside precursors and derivatives of purine analogs (with drug names and related IUPAC nomenclature) used or under investigation in the HCoV disease therapy.

Among the synthetic drugs under scrutiny in the treatment of viral respiratory pathologies, nucleoside precursors occupy an important place, especially in the present COVID-19 pandemic [54]. Here below, we report on the main nucleoside precursors evaluated as anti-HCoV drugs (Figure 1).

### 5.1. Favipiravir

Favipiravir (also indicated as T-705, **1**, Figure 1) is an oral drug that inhibits the RdRp of a wide range of RNA viruses including influenza, rhinoviruses, and also non-respiratory RNA viruses [96,97]. Its use was previously approved for influenza in Japan and as an experimental drug for Ebola virus infection [98]. However, the recent interest paid to this drug originates from the reports of favipiravir efficacy in COVID-19 management. 

Even though the matter is still controversial, its utility in prevention of pneumonia and SARS aggravation was suggested by randomized studies on COVID-19 patients treated with this safe and well-tolerated drug, who achieved SARS-CoV-2 viral clearance in 62.5% of cases within four days from the favipiravir treatment [99]. In another study, SARS-CoV-2-positive patients in severe or critical conditions who were administered this drug for their deteriorating conditions were eventually cured [100].

**Figure 2:**
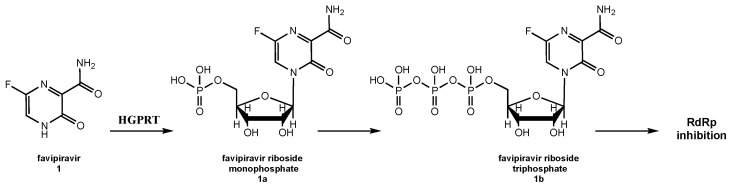
Mechanism of action of favipiravir.

Owing to its mechanism of action, intracellularly, **1** undergoes its hypoxanthine-guanine phosphoribosyltransferase (HGPRT)-mediated conversion into mono-(**1a**) and, subsequently, triphosphoribosylated (**1b**) form, which is recognized as a substrate by the viral RdRp, acting as a chain terminator and, thus, inhibiting the viral polymerase activity (Figure 2) [101]. 

### 5.2. Oxypurinol 

Oxypurinol (also known as XORTX, **2**, Figure 1) is an inhibitor of xanthine oxidase [102] that is known to be effective for decreasing the production of uric acid, an aspect relevant especially in medical conditions like gout and kidney stones, but also for preventing inflammatory responses [103,104]. Due to its potential preventive activity against the SARS hyperinflammatory response, oxypurinol was proposed in combination with pentoxifylline (**3**, Figure 1) to treat COVID-19 [105].

### 5.3. Pentoxifylline

Pentoxifylline (also referred to as oxpentifylline, **3**, Figure 1), a synthetic nucleoside precursor structurally related to caffeine and theophylline, in combination with oxypurinol, is able to reduce the effects of experimentally-induced inflammation [106]. In fact, pentoxifylline inhibits the production of TNF-α, a receptor that activates inflammatory genes such as IL-6, which regulates neutrophil infiltration into the interstitial lung tissue, causing severe injuries [105]. Since pentoxifylline may also exert its anti-inflammatory action in the hyperinflammatory phase in COVID-19 patients, it was hypothesized that early treatment of SARS-CoV-2-infected individuals with pentoxifylline, also combined with oxypurinol, could be a valid preventive anti-SARS treatment [105]. Both **2** and **3** are licensed drugs already in use for therapy of different diseases, and, consequently, this would avoid phase I clinical trials for their administration to COVID-19 patients [105].

### 5.4. Derivatives of Purine Analogs

Even if it cannot be properly considered a nucleoside precursor, baricitinib (the active ingredient of Olumiant drug, **4**, Figure 1) is a purine derivative previously known as a Janus kinase inhibitor and approved for the treatment of rheumatoid arthritis [107]. It was recently used in COVID-19 therapy combined with remdesivir, but its effectiveness is still a matter of debate [108]. Moreover, methotrexate (**5**, Figure 1), a well-known anticancer and immune suppressant drug [109], was recently investigated as a potential anti-COVID-19 drug and proved effective in inhibiting the SARS-CoV-2 virus replication in vitro [110].

## 6. Nucleoside Analogs as Anti-HCoV Drugs

**Figure 3:**
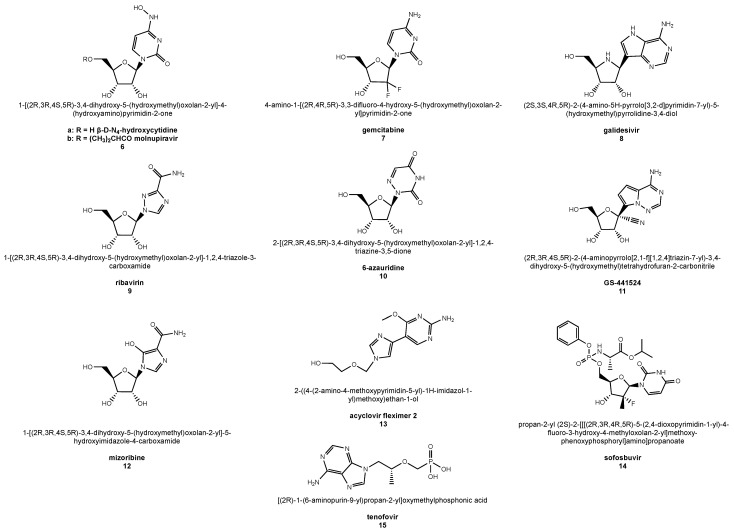
Structures of the nucleoside analogs (provided with drug names and related IUPAC nomenclature) used or under investigation in the HCoV disease therapy.

Nucleoside analogs are widely used as drugs against infections caused by herpes viruses, hepatitis B virus, human immunodeficiency virus (HIV) but also against RNA virus infections such as hepatitis C [62]. The main advantages of nucleosides over non-nucleoside antiviral agents include their applicability to a broad-spectrum of viral strains or species and their ability to overcome the antiviral resistance [62]. This is due to the polymerase inhibitor mechanism of action common to the majority of nucleoside analogs used in the antiviral therapy and to the well-conserved nature of nucleotide-binding sites in polymerases among the different virus families [111]. In detail, intracellularly, most nucleoside analogs are converted by host kinases to their triphosphate forms, which compete with natural nucleoside triphosphates in binding to the viral polymerase active site, eventually impairing RNA or DNA synthesis [112]. Structurally, nucleoside polymerase inhibitors are both purinic and pyrimidinic synthetic derivatives in which the sugar or the nucleobase underwent chemical modification (Figure 3). Administered as nucleotide precursors, they are metabolized by kinase enzymes to the active triphosphate forms inside the host. The unnatural nucleotides are then incorporated into replicating viral genomes leading to chain termination and subsequent blockage of replication or transcription [112]. In another mechanism, nucleoside analogs are incorporated into elongating nucleotide chains, potentially leading to mutations that alter one or more fundamental viral processes such as RNA structuring, its interaction with proteins, viral protein functions, and RNA synthesis [113]. As mentioned, nucleoside analogs overcome the antiviral resistance because the binding site of their polymerase targets is highly conserved among virus families, with amino acid conservation percentages as high as 70–100% in the case of CoVs [113]. This highlights the importance of developing nucleoside analogs as anti-coronavirus drugs with RdRp inhibitory activity. Here below, we summarize the properties of some of the most relevant drugs belonging to this family of synthetic HCoV inhibitors (Figure 3).

### 6.1. β-d-N4-Hydroxycytidine

*β*-d-*N*4-Hydroxycytidine (NHC, **6a**, Figure 3) is a cytidine analog with broad-spectrum antiviral activity as found with influenza A and B viruses, respiratory syncytial virus, Venezuelan equine encephalitis virus, zoonotic group 2b or 2c bat-CoVs [114], and HCoVs [115]. This drug’s mechanism of action is based on NHC-triggered mutagenesis of viral RNA and, as found in the case of HCoVs, on its interaction with viral replicase. NHC showed potent antiviral activity against the coronaviruses HCoV-NL63 and, more interestingly, SARS-CoV-1 [115]. In vitro studies also demonstrated the ability of NHC to inhibit MERS-CoV with minimal cytotoxicity [116], and its applicability was also proposed for COVID-19 therapy [114].

### 6.2. Molnupiravir

Molnupiravir (also known as MK-4482 and EIDD-2801, **6b**, Figure 3) is a prodrug of **6a** active as an antiviral drug against influenza [117]. This nucleoside analog blocked SARS-CoV-2 transmission in animal models [118] and was used in one-year stage II/III trial on hospitalized COVID-19 patients [119].

### 6.3. Gemcitabine 

Gemcitabine (**7**, Figure 3) is a deoxycytidine analog with a broad-spectrum antiviral activity that inhibits SARS-CoV-1 and MERS-CoV [120]. Gemcitabine potential as an anti-COVID-19 drug was recently investigated in cell culture [121]. This nucleoside analog inhibited SARS-CoV-2 replication in infected Vero-E6 cells at noncytotoxic drug concentrations [121]. In the same study, cytidine nucleoside suppressed the gemcitabine antiviral effect while, combined administration of gemcitabine with oxysophoridine led to an additive antiviral effect against the coronavirus [121].

### 6.4. Galidesivir

Galidesivir (also known as BCX4430 or Immucillin-A, **8**, Figure 3) is an adenosine analog with broad antiviral activity against several RNA viruses and low cytotoxicity [122]. In vitro, **8** exerts antiviral activity against pathogenic HCoVs with coronavirus inhibition observed in cells infected with SARS-CoV-1 and MERS-CoV [122]. At the same time, in silico, **8** shows potent activity against SARS-CoV-2 due to its tight binding to the HCoV RdRp [123].

### 6.5. Ribavirin 

Ribavirin (**9**, Figure 3), also referred to as Virazole or ICN 1229, is an analog of guanosine used as a broad-spectrum antiviral drug against infections of different RNA viruses, including hepatitis C virus (HCV), hantavirus, and respiratory syncytial virus [124,125]. Two main mechanisms for its antiviral action were described involving the drug in monophosphate and triphosphate form, respectively. The first form interacting with the host nucleotide-synthesizing enzyme IMPDH (inosine monophosphate dehydrogenase) lowers the guanosine production levels with consequent inhibition of viral RNA synthesis [124]. In the second mechanism, ribavirin triphosphate incorporated by the viral polymerase provokes lethal mutagenesis [124]. However, the coronavirus exonuclease proofreading activity shields HCoVs from ribavirin antiviral action, even using drug doses that proved active against other RNA viruses [113]. Interestingly, at high doses, this guanosine analog partially inhibited in vitro replication of MERS-CoV and SARS-CoV-1, leading, however, to disease worsening in animal models of SARS-CoV-1 infection [113]. Remarkably, no significant clinical benefit was revealed in patients affected by SARS who experienced even drug toxicity [113]. Though the combined treatment with well-tolerated doses of ribavirin and interferon IFNα2b resulted in an increased therapeutic effect of the nucleoside drug, in both in vitro and animal models of MERS-CoV disease [126], when the same approach was applied to human subjects with critical MERS-CoV infection, no efficacy was recorded [113]. Moreover, survival was not improved in MERS patients receiving **9** with both interferons IFNα2a and IFNβ1a [113]. Ribavirin found interest in the experimental therapy of COVID-19, also in combination with other antiviral agents and interferon IFNβ1b [76], following evidence of its in vitro efficacy on SARS-CoV-2 [75]. However, although this guanosine analog shows anti-HCoV efficacy in vitro, there is no agreement on its clinical benefit to patients with SARS-CoV-1, SARS-CoV-2, or MERS-CoV infections.

### 6.6. 6-Azauridine

The uridine analog 6-azauridine (**10**, Figure 3) is endowed with orotidine monophosphate decarboxylase inhibitory activity and consequent antiviral effect [127]. It was effective against HCoV-NL63 at the early stages of HCoV replication [128] and inhibited replication of SARS-CoV-1 at non-toxic doses [127]. Although no experimental data on its effect on SARS-CoV-2 are currently available, this nucleoside analog is among the drugs to be considered for COVID-19 treatment [129].

### 6.7. GS-441524

**Figure 4:**
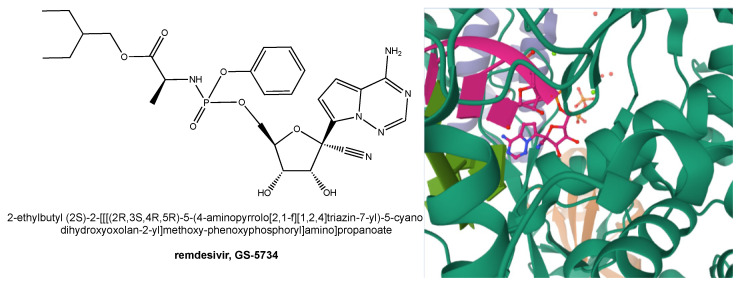
Structural representation of remdesivir (with use and IUPAC names, **left**) and view of its triphosphate form bound to SARS-CoV-2 RdRp in the active site (PDB ID: 7BV2 [132], image obtained using Mol* [133], https://www.rcsb.org/3d-view/7BV2 (accessed on 10 December 2020), **right**).

Gilead Science (GS)-441524 (**11**, Figure 3) is a 1′-cyano adenosine analog, which is the main plasma metabolite of the more famous antiviral drug remdesivir (GS-5734, Figure 4) [130]. Several cellular studies conducted on **11** indicated an anti-SARS-CoV-2 activity comparable when not higher than remdesivir [131], with some studies pointing out that **11** would be even more convenient than remdesivir for the COVID-19 therapy [131]. GS-441524 advantages over remdesivir include ease of synthetic preparation, lower hepatic toxicity, as well as oral administration route (not suitable for remdesivir due to its poor liver stability) [131].

### 6.8. Mizoribine

Mizoribine (also known as MZB, **12**, Figure 3) is an imidazole nucleoside with known inosine-5’-monophosphate dehydrogenase inhibitory activity [134]. Mizoribine inhibited the replication of SARS-CoV-1 more effectively than ribavirin, but it could not completely inhibit the replication of the virus even at concentrations up to 100 µg/mL [135]. As an inhibitor of the proinflammatory protein HSP60, **12** was recently identified as an already clinically approved drug able to potentially ameliorate severe inflammatory reaction in COVID-19 patients [136].

### 6.9. Acyclovir Fleximer 2

A flexible-base modification of the guanosine analog acyclovir (herein indicated as acyclovir fleximer 2, **13**, Figure 3) showed activity against HCoV-NL63 in cellular studies [137]. The same derivative could block the replication of the highly pathogenic MERS-CoV but not SARS-CoV-1, while unmodified acyclovir had no effect under the same experimental conditions [137]. In our opinion, this compound and the entire family of acyclovir fleximers merit further investigational efforts in the fight against COVID-19.

### 6.10. Nucleotide Drugs: Sofosbuvir and Tenofovir

Sofosbuvir (**14**, Figure 3) is a nucleotide analog used to treat HCV infection, which can inhibit the SARS-CoV-1 RNA-dependent RNA polymerase [138], affecting the viral life cycle, as underlined in the study of Li and De Clercq [139]. Together with the acyclic nucleotide phosphonate (ANP) tenofovir (**15**, Figure 3) [140], sofosbuvir was predicted to bind the SARS-CoV-2 RdRp tightly, blocking the viral life cycle at the stage of nucleotide incorporation [140]. This mechanism was experimentally proven by Chien et al., who demonstrated that SARS-CoV-2 RdRp can incorporate the triphosphate forms of sofosbuvir and tenofovir, thus blocking the incorporation of further nucleotide triphosphates [141]. On the other hand, the studies performed by the group of Schinazi showed no antiviral effect for sofosbuvir against SARS-CoV-2 [142]. In contrast, a randomized controlled trial on COVID-19 patients showed that adding this drug to standard care significantly reduced hospital stay duration but did not have any statistically-significant benefit on mortality [143]. In regard to tenofovir, no published data from randomized trials are presently available, and only a few clues of its protective role against SARS-CoV-2 infection emerged by a preliminary clinical study [144]. 

RdRp represents the main target of anti-HCoV nucleoside drugs. Though the alignment of the amino acid sequences of the RdRp found in the three highly pathogenic coronaviruses revealed high homology and conservation rates, in close analogy with other positive-sense RNA viruses (like HCV [145]), among HCoVs SARS-CoV-2 RdRp shares 96% homology with SARS-CoV-1 RdRp, but only 70% with MERS-CoV RdRp [146]. MERS RdRp structure has not yet been solved experimentally. Still, attempts were made to describe it computationally [147]. The results suggested that, despite the lower overall homology levels of MERS-CoV RdRp, its active site is still highly conserved within the positive-sense RNA viruses. Therefore, HCV RdRp inhibitors may also be effective inhibitors of MERS-CoV RdRp [147]. On this basis, sofosbuvir, developed as an HCV RdRp inhibitor, could be repositioned to combat all three the epidemic/pandemic-causing HCoVs.

## 7. Remdesivir as an Anti-HCoV Drug and Its Role in the Fight against COVID-19

**Figure 5:**
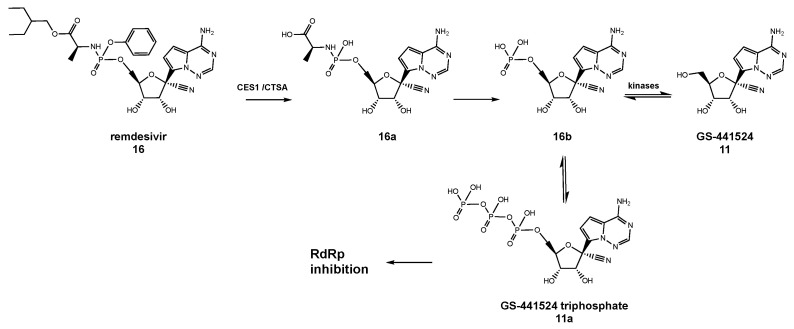
Mechanism of the RdRp inhibitory activity of remdesivir and its non-phosphorylated nucleoside form GS-441524.

Remdesivir, also known as Gilead Science GS-5734 (**16**, Figure 4), is a phosphoramidate prodrug of the above-described nucleoside analog **11**, which inhibits several families of viruses ranging from pneumoviruses, filoviruses, paramyxoviruses, and Ebola virus to human and zoonotic CoVs [130]. Owing to its mechanism of action (Figure 5), **16** intracellularly is presumed to undergo conversion to its triphosphate form **11a**, which acts as a non-obligate chain terminator in viral RNA synthesis. In more detail, upon host cell entry, **16** is hydrolyzed by enzymes carboxylesterase 1 (CES1) and cathepsin A (CTSA) to a carboxylate form followed by loss of the phenoxide moiety to yield the derived alanine metabolite **16a** (Figure 5) [148].

This metabolite subsequently undergoes hydrolysis to the monophosphate form **16b**, which is in turn hydrolyzed to GS-441524 (**11**) or phosphorylated by cellular kinases leading to the triphosphate form (**11a**) active in RdRp inhibition (Figure 5).

Remdesivir acts as a potent inhibitor of replication of MERS-CoV and SARS-CoV-1 in multiple in vitro models. Moreover, in animal studies, **16** displayed protective effects on mice infected with SARS-CoV-1 [149]. Since **16** had previously shown promise in the managing of socially-relevant viral diseases, and following the previously-emerged clues of efficacy also against HCoVs, this drug was also early considered for COVID-19 therapy. FDA granted its emergency use authorization as an investigational drug for the treatment of COVID-19 on 1 May 2020 [150]. The encouraging results that came from randomized controlled trials led the same agency to approve on 22 October 2020 the administration of **16** (under the brand name Veklury) to 12 years of age and older COVID-19 patients for the treatment of the SARS-CoV-2-associated disease [82]. After that date, different meta-data analyses were published based on results from several clinical trials investigating the drug’s role in COVID-19 therapy, confirming at least partially the initial enthusiasm for this antiviral treatment. Primary outcomes taken into consideration in these data-analyses were the recovery and mortality rates, while secondary ones were the drug safety profile. One of these studies [151], based on one observational study and four clinical trials, showed that the recovery rate on day 14 among severe COVID-19 patients treated with **16** for ten days increased by 50%, while a 14% increase was recorded on day 28 among the moderate and severe group [151]. Moreover, the mortality rate on day 14, but not on day 28, was found decreased by 36% among all the remdesivir-receiving patients. Both responses to drug and mortality rates were more favorable in nonmechanically ventilated patients. Another meta-analysis study [152] compared COVID-19 patients receiving 10-day remdesivir treatment with placebo/standard of care groups, showing that the drug significantly reduced not only the 14-day mortality but also the requirement for mechanical ventilation, as well as severe adverse effects. Other indicators such as clinical improvement on day 28, day 14 clinical recovery, and day 14 discharge rate were found significantly more favorable in the same study among the remdesivir-receiving group. Earlier clinical improvement and recovery were observed among the patients who received **16**. When comparing 10-day and 5-day courses of **16**, the longer course was accompanied by a higher discharge rate at day 14 and higher rates of serious adverse effects [152]. 

## 8. Remdesivir Adverse Side Effects

Although remdesivir has shown promise as a treatment for COVID-19, recent studies have raised concerns about potential adverse drug effects. In general, the more adverse outcome is observed in a longer course of the drug [152], but these are generally no significant grade III or IV adverse effects [151].

Apart from the more common low-grade side effects occurring during or soon after the time of drug infusion (such as low blood pressure, nausea, vomiting, and sweating [153]), marked sinus bradycardia, occurring acutely on the initiation of drug therapy and resolving almost immediately on its cessation, was reported [154]. An accurate study on the adverse drug events (ADEs) associated with remdesivir administration to COVID-19 patients analyzed 439 cases that reported 1004 adverse effects [155]. These ADEs, classified as severe in more than 80% of cases, were mainly from male patients older than 45 and corresponded mostly to: rise in hepatic enzyme levels, kidney injury, increased creatinine levels, and respiratory failure [155]. Overall, deterioration of hepatic, renal, and, less frequently, cardiac function were observed as ADEs associated with remdesivir administration, highlighting the need to monitor remdesivir-receiving patients for these ADEs carefully.

## 9. Conclusions

In this review, we summarized the most relevant literature regarding the development of nucleoside precursors and analogs as treatments for coronaviruses infections, with particular attention paid to the most pathogenic SARS-CoV-1, MERS-CoV, and especially SARS-CoV-2, which is at the origin of the current COVID-19 pandemic. We found that one of the main effects on the HCoV life cycle provoked by both classes of molecules is the competitive inhibition of the RdRp-mediated viral RNA synthesis. The triphosphate form of nucleoside derivatives is incorporated by RdRp, preventing the incorporation of additional nucleotides. In contrast, nucleoside precursors are initially converted into their ribonucleotide forms that successively undergo the same fate (Figure 2).

The low overall human-to-human transmission potential of MERS [156] and the disappearance of SARS in 2004 [74] may explain the low scientific interest paid to discover effective prophylactic and therapeutic weapons against HCoVs before the current COVID-19 era. This gap became evident when in late 2019 SARS-CoV-2 emerged in Wuhan, causing what eventually revealed the current pandemic. The urgent demand for an effective anti-COVID-19 therapy led to examining ‘old drugs’ already in use for other pathology treatments, whose safety profile had already been assessed. Among the several repurposed drugs under investigation against SARS-CoV-2, nucleoside analogs lately conquered a central role. Of these, the uridine monophosphate analog sofosbuvir, the modified cytidine molnupiravir, the adenosine analog GS-441524, and its monophosphate prodrug remdesivir showed encouraging results, justifying the recent FDA approval of this latter drug as the only specific anti-COVID-19 therapeutic agent available to date [82]. Even though remdesivir can be safely administered to hospitalized COVID-19 patients, it shows its optimal effect on those who are severely affected but not mechanically ventilated [151], leaving still unsolved the issue of treating the other cases by a specific anti-SARS-CoV-2 therapeutic agent. In our opinion, this gap could be mitigated through systematically evaluating the anti-COVID-19 effects of other nucleoside analogs or nucleoside precursors, including those listed in this review, for most of which only predictive studies, and no experimental data, on their ability to inhibit SARS-CoV-2 are available to date. As with SARS-CoV-1, MERS-CoV, and SARS-CoV-2, in the future, new zoonotic CoVs will likely be released from the heterogeneous virus pools present in animal reservoirs, and this could lead to future global threats. Therefore, it is fundamental to develop new broad-spectrum anti-HCoV prophylactic and therapeutic strategies aimed at multiple biomolecular targets and functions conserved across the HCoVs. More research into nucleoside/nucleoside precursor development, also starting from new synthetic structures, as well as their evaluation as HCoV antivirals, is urgently needed, and the scientific research in this field should not be interrupted when, hopefully soon, the current pandemic will be over.
